# Occurrence of sporotrichosis in Belém, Pará, Brazil: a metaphor for unsustainable socioeconomic development

**DOI:** 10.1016/j.bjid.2024.103872

**Published:** 2024-09-24

**Authors:** Nelson Veiga Gonçalves, Claudia do Socorro Carvalho Miranda, Bruna Costa de Souza, Matheus Pereira do Couto Rocha, Francisca Regina Oliveira Carneiro, Marcelino Antônio Costa Maués, Déborah Mara Costa de Oliveira, Maridelzira Betânia Moraes David, Mioni Thieli Figueiredo Magalhaes de Brito, Juarez Antonio Simões Quaresma

**Affiliations:** a Universidade do Estado do Pará, Laboratório de Epidemiologia e Geoprocessamento da Amazônia, Belém, PA, Brazil; b Universidade Federal Rural da Amazônia, Instituto Ciberespacial, Belém, PA, Brazil; c Universidade Federal do Pará, Instituto de Ciências da Saúde, Belém, PA, Brazil; d Secretária Municipal de Saúde de Belém, Centro de Controle de Zoonozes, Belém, PA, Brazil; e Universidade Federal Rural da Amazônia, Hospital Veterinário, Belém, PA, Brazil

**Keywords:** Sporotrichosis, Living conditions index, Public health, Spatial analysis

## Abstract

Sporotrichosis is a fungal anthropozoonosis that has become a major public health problem in tropical countries. With that in mind, this study analyzed the relationship between this disease and demographic, socioeconomic and public health issues in Belém, State of Pará, Brazil, from 2020 to 2022. This ecological and cross-sectional study used data from the Belém Zoonosis Control Center, the Brazilian Institute of Geography and Statistics and the Health Ministry. Descriptive and spatial analyses were performed employing significance statistical, kernel, buffer and Moran techniques. One hundred sporotrichosis cases in cats and 49 in humans were analyzed. The results showed that the individuals most affected were women (61.22 %), adults (87.76 %), with the cutaneous form (95.92 %), diagnosed histopathologically (38.78 %), still undergoing treatment (46.94 %) and that the form of contagion was through cat scratches or bites (73.47 %). The profile also showed quantitative significance of ignored data related to treatment (65.31 %) and cat presence at home (63.27 %). The disease had a non-homogeneous distribution with very high densities in Campina de Icoaraci, Águas Negras and Parque Guajará. Those neighborhoods presented a very low Living Conditions Index and precarious services and health centers. The spatial dependence between the environmental and socioeconomic studied variables evidenced the establishment of an active transmission circuit for sporotrichosis in peripheral areas of the city, related to health inequalities with an underlying possible epidemiological silence, suggesting the need for expanding One Health public policies, aiming the sustainable development.

## Introduction

Lutz and Splendore^1^ carried out the first Brazilian studies focusing on the infection of animals by fungi of the genus Sporothrix. Sporotrichosis is the mycotic disease that results from this infection and can affect several animal species whose interaction with human populations can lead to their illness, making this disease an anthropozoonosis. The main risk factors are principally related to injuries caused by biting and scratching by domiciled or abandoned infected cats, which are the main hosts.^2^^,^^3^ The disease has several clinical forms, ranging from a single lesion to systemic and disseminated forms that can affect the lungs, mucous membranes, meninges, bones and joints.^4^

The occurrence of this anthropozoonosis in both humans and cats, has a worldwide distribution with higher prevalence in temperate and tropical regions, where the fungus finds the most desirable conditions for development and is endemic in South Africa, India, China, Japan, and Latin America.^5^^,^^6^ In Brazil, sporotrichosis is considered emerging with a significant number of cases in the metropolitan state capital areas of the south and southeast and a tendency to expand to the rest of the country during the last two decades.^5^^,^^6^ Because there is no protocol for compulsory notification of this disease in Brazil, which is only required in the states of Rio de Janeiro and Pernambuco, its epidemiological scenario is still unknown in the Brazilian territory as a whole. This poses a problem for public health related to its spatial and temporal pattern of occurrence, mainly because of its possible relationship with demographic, socioeconomic, environmental and public health variables as observed in other infectious diseases.^5^^,^^7^^,^^8^

In 2018, sporotrichosis cases were identified in more than eight states in the Southeast region of Brazil, mainly in the states of Rio de Janeiro, Minas Gerais, and São Paulo. That year, the northern Brazilian city of Belém do Pará with 1.360.167 inhabitants reported the first cases of the disease in both humans and animals.^9^ However, in this region, there are still few reports of the disease, although its risk factors such as favorable climatic variables and socioeconomic issues are observed. The city has seven contiguous Administrative Districts (AD): Belém (ADBEL); Outeiro (ADOUT); Entroncamento (ADENT); Guamá (ADGUA); Sacramenta (ADSAC); Benguí (ADBEN) and Icoaraci (ADICO). In this territorial configuration, the ADBEL is formed by neighborhoods located in the central area with the others in the peripheral ones. These districts are marked by great differences in sanitation infrastructure, population distribution, and socioeconomic level, related to the disorderly territorial expansion that has occurred in the city over the last few decades.

The differential conjunction of the demographic, environmental, and socioeconomic characteristics related to the distribution of living conditions index for the population associated with sanitary and environmental variables in the city neighborhoods and administrative districts may thus be producing risk factors for the occurrence of the disease based on the logic of socio-spatial segregation of the population. Therefore, analyzing the distribution of sporotrichosis in this territory carried out within the scope of epidemiological surveillance is a major challenge for studies in public health, since this scenario observed in Belém is recurrent in other Brazilian cities as well.

Because they are able to use logical and mathematical representations of geographical areas, geoprocessing techniques have been widely used to analyze the spatial dependence of the occurrence of various anthropozoonoses and their relationship with the living conditions of human populations and the provision of health services in Brazil according to its demography. This is in accordance with Law 8080 of September 1990, which created the Unified Health System in compliance with the principle of equity.^10^, ^11^, ^12^ The use of spatial data analysis techniques in health has thus enabled the development of studies based on etiological hypotheses, including the identification of areas with active transmission circuits of infectious diseases and their underlying conditions.

These techniques have recently also been used to highlight the production of socio-spatial relationships in territories based on the hierarchical identification of access to health services and its association with the occurrence of diseases, especially those that are produced by environmental and socioeconomic issues such as some anthropozoonoses, e.g., sporotrichosis. Given the need to develop an epidemiological scenario for this disease, this study sought to analyze its relationship with demographic, socioeconomic, and public health policy variables in the city of Belém, the capital of Pará state, for the period from 2020 to 2022.

## Materials and methods

This ecological and cross-sectional study analyzed the distribution of all laboratory-confirmed sporotrichosis cases in Belém, Pará, Brazilian eastern Amazon, from January 2020 to December 2022, considering its contiguous neighborhoods and administrative districts as spatial units for analysis.

Epidemiological data on the human cases (Gender, Age Group, Evolution, Form of Contagion, Clinical Presentation, Diagnosis Exam, Treatment and Cat Presence in the Home) and the location of infected cats were obtained from the sporotrichosis report produced by the Zoonosis Control Center (CCZ) of the Belém Municipal Health Secretariat. Data regarding the health centers and their services were acquired from the National Registry of Health Establishments at the Ministry of Health. The Living Conditions Index (LCI) and Demographic data were used, based on the estimation process for 2020, and from the 2010 Census carried out by the Brazilian Institute of Geography and Statistics. The cartographic data and satellite images were obtained from the National Space Research Institute.

Next, the data obtained were debugged to remove incomplete or inconsistent records using Tabwin 36b software. They were subsequently georeferenced in the laboratory using Google Earth Pro in order to build a Geographical Database. In the descriptive analysis, the proportion calculus and the non-parametric chi-squared statistical test of expected equal proportions for the individual profiles were performed considering a *p*-value of <0.05 as significant, as well as the quartile intervals of the demographic data (Low, Moderate, High and Very High). Biostat 5.4 software was used for that purpose.

In order to calculate the LCI an adaptation of the work of Gonçalves^13^ and Paim^14^ was made, using the following socioeconomic indicators: income (proportion of heads of private households with an average monthly income equal to or less than two minimum wages); education (proportion of literate people aged 10 to 14 years); cluster (proportion of houses in subnormal agglomerations, such as slums and squatter settlements in relation to the total number of households); rm/q (ratio between the average number of residents per household and the average number of bedrooms) and sanitation (percentage of households with internal plumbing connected to the water supply network).

The LCI was calculated for each neighborhood in the city, considering the average values of income, cluster and rm/q, which were arranged in ascending order and those of education and sanitation in descending order. Next, each one received an ascending score, relative to the value of each indicator. In order to obtain the LCI values (lowest value of 20.33 and highest value of 44.32), the sum of the scores of the five indicators of each neighborhood was performed and grouped into quartiles so that they could be classified into strata of life conditions: high (from 20.33 to 27.79), moderate (from 27.80 to 34.50), low (from 34.51 to 39) and very low (from 40 to 44.32). Lower LCI values corresponded to better living conditions. In an effort to verify the precarious living conditions of the resident populations in peripheral areas of the city, four fieldwork activities were carried out.

Several techniques were used for the spatial analysis, such as kernel density estimation to evaluate the distribution of human sporotrichosis, considering the distance between the geographic points of the occurrence of the cases, and a buffer to estimate the ecological niche coverage for infected cats, calculating the radius of displacement of these animals to meet their physiological demands. The bivariate local Moran Index (I) was also used to verify the spatial autocorrelation, between areas with cases in both humans and animals. This was considered direct for I > 0, inverse for I < 0, and strong when indices were close to one of the defined variation limits (−1 and 1).^10^ The analyses of the relationships between the variables and their expressions in thematic maps were performed using Arcgis 10.5 software.

The present study was exempted by the Research Ethics Committee of the Health Science Institute at the Universidade Federal do Pará (Pará Federal University) under opinion n° 1.684.124/2016 and by the Ethics Committee on the Use of Animals, of the Universidade Federal Rural da Amazônia (Federal Rural University of the Amazon) under protocol n° 1464170522, both in accordance with Resolution n° 466/12 of the National Health Council and with law n° 11.794, of October 8, 2008.

## Results

Regarding the quartile calculation of the district population quantitative of Belém, the following distribution was observed: low in ADOUT, moderate in ADENT and ADBEL, high in ADICO and ADSAC, and very high in ADGUA and ADBEN. The districts that most presented cases in humans and animals were ADICO and ADBEN, which are spatially contiguous. In relation to the occurrence of the disease in felines, one hundred cases in different AD and neighborhoods of the city were analyzed. The epidemiological profile analysis of the forty-nine cases of human sporotrichosis showed a major occurrence of the disease in women (61.22 %), adults (87.76 %), with cutaneous form (95.92 %), diagnosed histopathologically (38.78 %), still undergoing treatment (46.94 %) and form of contagion by cat scratches or bites (73.47 %). The profile also showed a quantitative significance with ignored data related to treatment (65.31 %) and cat presence at home (63.27 %). All variables were significant except for gender and the diagnosis exam (Table 1). The pathogen identified as responsible for the infection was *Sporothrix schenckii*.

**Table 1:** Epidemiological profile of human sporotrichosis, in Belém, Pará State, Brazil, from 2020 to 2022.

Variable	Category	Frequency (n = 49)	Proportion (%)	*P-value
Gender	Female	30	61.22	0.2120
	Male	19	38.78	
Age group	Adolescent	1	2.04	< 0.0001
	Adult	43	87.76	
	Elderly	4	8.16	
	Ignored	1	2.04	
Form of Contagion	Cat Scratches/Bites	36	73.47	< 0.0001
	Contact with Suspected Animal	11	22.45	
	Ignored	2	4.08	
Evolution	Discharged	2	4.08	< 0.0001
	Cure	7	14.29	
	Undergoing Treatment	23	46.94	
	Ignored	17	34.69	
Clinical presentation	Cutaneous Form	47	95.92	< 0.0001
	Ignored	2	4.08	
Diagnosis exam	Fungal Culture	13	26.53	0.5647
	Histopathological	19	38.78	
	Ignored	17	34.69	
Treatment	Itraconazole	16	32.65	< 0.0001
	Spontaneous Healing	1	2.04	
	Ignored	32	65.31	
Cat presence in the home	Yes	17	34.69	< 0.0001
	No	1	2.04	
	Ignored	31	63.27	

n, Number of cases;

⁎ Adherence chi-square, *p* < 0.05.

**Fig. 1:**
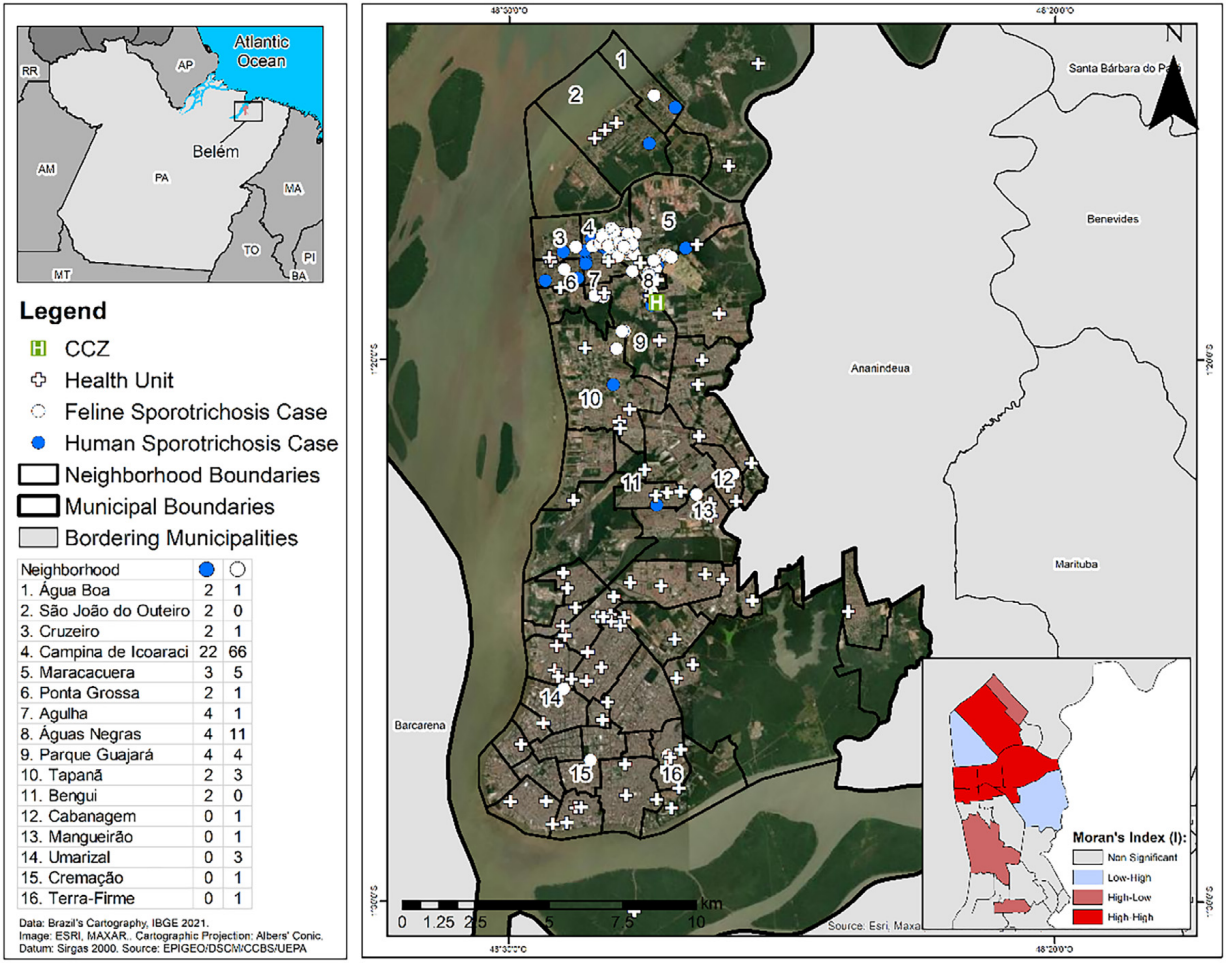
Spatial distribution of human and feline sporotrichosis and health units, in the neighborhoods of Belém, Pará State, Brazil, from 2020 to 2022.

An overall non-homogeneous spatial distribution of sporotrichosis in both humans and animals was observed in the studied area for the period. The highest numbers for the disease in humans were verified in the neighborhoods of Campina de Icoaraci (22), Águas Negras (4), Parque Guajará (4) and Agulha (4). Regarding the infected cats, the highest occurrence was observed in Campina de Icoaraci (66), Águas Negras (11) and Maracacuera (5). The bivariate local Moran Index (I) showed a direct and strong “High-High” spatial autocorrelation, with statistical significance between the occurrence of the disease in humans and animals in the neighborhoods listed above, as well as in Cruzeiro and Ponta Grossa, all belonging to the ADICO, and São João do Outeiro in the ADOUT. All these contiguous neighborhoods are located in peripheral areas of the city (Fig. 1).

It was also observed that the provision of health services and establishments related to environmental and epidemiological surveillance presented a significant precariousness in the peripheral areas of the city. This situation was principally associated with the low coverage of public laboratories for the diagnosis and availability of endemic combat agents, especially in all ADICO neighborhoods, these being Águas Negras, Agulha, Campina de Icoaraci, Cruzeiro, Maracacuera, Paracuri, Parque Guajará, Ponta Grossa and Tenoné (Fig. 1).

**Fig. 2:**
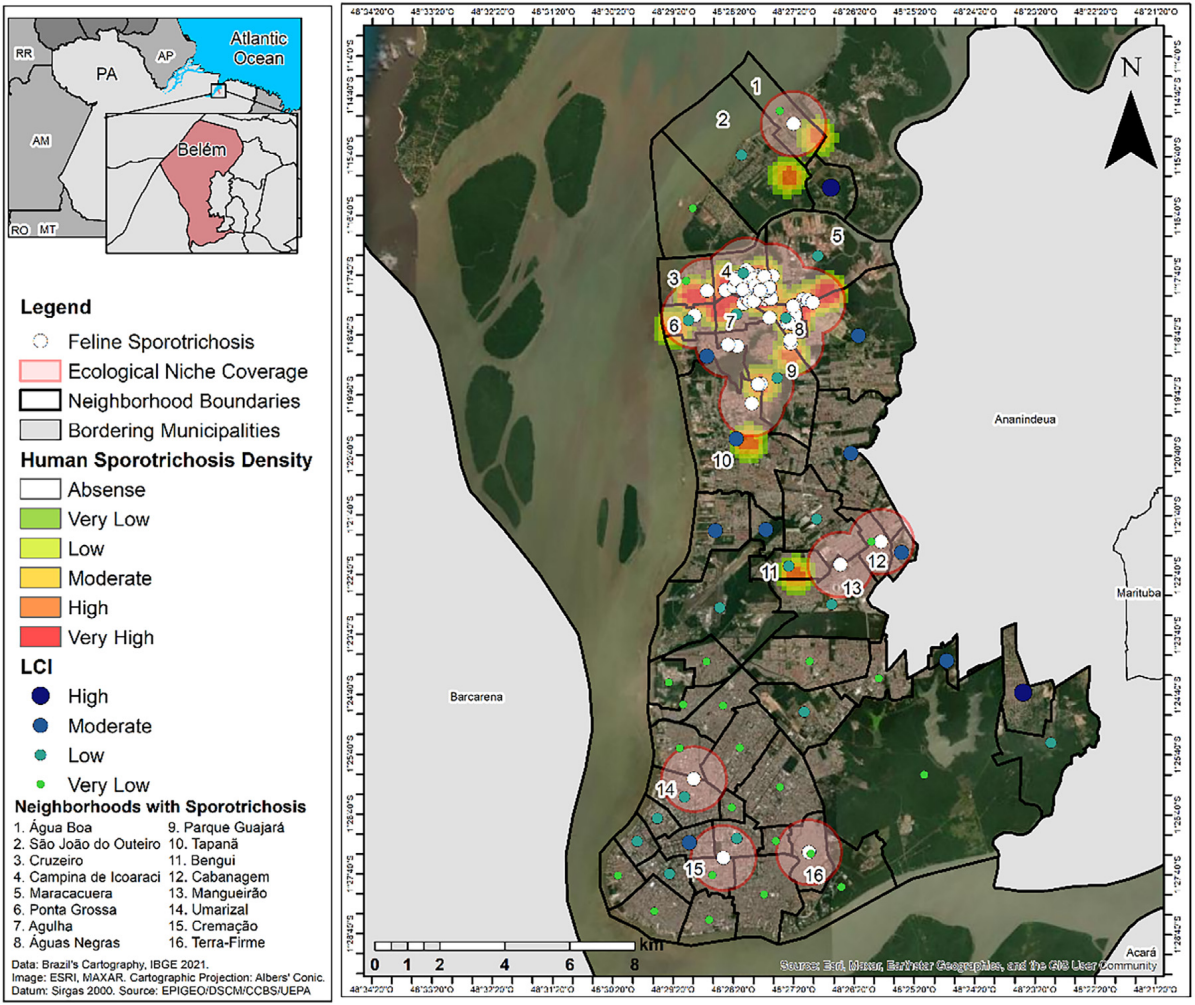
Density of human cases of sporotrichosis and living conditions index (LCI), in the neighborhoods of Belém, Pará State, Brazil, from 2020 to 2022.

Analysis of the sporotrichosis cases in humans performed with the kernel estimation technique showed densities that were very high in the Cruzeiro, Campina de Icoaraci, Parque Guajará, Ponta Grossa, Agulha and Águas Negras neighborhoods, high in Maracacuera and Tapanã, and moderate in Água Boa, São João do Outeiro and Bengui. It was also observed that the areas covered by the ecological niche with the largest number of infected cats, identified using the buffer technique are spatially dependent on the high density of the cases of the disease in humans (Fig. 2).

It was also identified that LCI in Belém presented a great socioeconomic difference between its neighborhoods, with a high index of living conditions identified in those located in the central area of the city, such as Nazaré, Batista Campos and Marco, and low or very low in peripheral ones, such as Água Boa, Cruzeiro, Bengui and Parque Guajará. In the neighborhoods that had a high LCI, there were no reports of sporotrichosis cases. However, in those where this indicator was low or very low, the disease density in humans was high or very high (Fig. 2).

The spatial dependence between the occurrence of the disease cases in both humans and cats observed with a non-random behavior using the kernel technique pointed to a geographic dependence between them, establishing a cluster of the disease. This scenario was confirmed by direct and strong spatial correlation between areas with high density of the disease in humans and those covered by the ecological niche of the infected felines, in Belém neighborhoods and AD, which presented precariousness in the offer of public health services and low living conditions. Thus, these observations highlight a process of socio-spatial segregation of the population in the studied area.

## Discussion

Considering that the first confirmations of human and animal sporotrichosis in Belém were only described in 2018,^15^ the number of cases analyzed during the studied period suggests the existence of an epidemiological silence possibly related to the non-mandatory notification of the disease in this state, as may be observed in several regions of Brazil.^5^^,^^8^ This situation may be also related with the significant number of infected cats in the various districts and neighborhoods of the city, as well as the presence of other risk factors for this pathology such as socioeconomic and environmental issues.^16^^,^^17^

The fact that sporotrichosis occurred in a higher percentage of adult women who reported contagion form by cat scratches or bites follows the national trend of the disease profile and may be related to their more frequent permanence in the home. This situation highlights that the coexistence between humans and domestic animals suggests a process of intra and peridomiciliary transmission of the disease due to these relationship risk factors, principally in peripheral areas.^18^^,^^19^ The high percentage of individuals undergoing treatment may be related to the recent beginning of the sporotrichosis diagnosis in the city, in addition to the therapeutic scheme adopted as a protocol, which is considered relatively long and expensive, especially when the case is diagnosed late. Thus, the difficulty in accessing laboratory tests and clinical follow-up for the disease makes for an inequity in health.^16^^,^^17^

The fact that the disease profile showed a quantitatively significant percentage of the cutaneous form as the main clinical presentation and ignored data from the diagnostic test and type of treatment indicates the need to expand the epidemiological surveillance for the disease. In this context, these three variables may indicate adverse consequences in relation to the disease cases growth, especially when the risk of a sporotrichosis outbreak may be imminent in Belém, notably when we consider the difficulty of access to laboratory tests and drugs such as itraconazole. The major amount of ignored data about cat presence in the home suggests the need to inform the primary health care professionals about its risk factors, as seen in others infectious processes.^20^^,^^21^

The significant number of cats with sporotrichosis, whose epidemiological process initiated at Campina de Icoaraci at ADICO in January 2020, may be related to the lack of contraceptive actions to control the large feline population of the city, principally in peripheral areas with a large number of abandoned animals.^17^^,^^22^ Another factor possibly associated with this situation is the growth of domestic cat breeding, which was observed recently during the COVID-19 pandemic.^1^^,^^2^^,^^23^ The high density of sporotrichosis cases in both humans and cats occurred in the Campina de Icoaraci, Parque Guajará and Águas Negras may be related to the socioeconomic characteristics of Belém peripheral areas, given its disordered urban expansion over the last decades with unsanitary occupations with low infrastructure and precarious public services, identified in these areas during fieldwork.

The geographical dependence observed between areas with human and feline sporotrichosis and LCI based on direct and strong spatial autocorrelation of the “High-High” type using Moran's local index technique, highlighted the social inequalities of the population resident in the ADICO neighborhoods, which was verified in the laboratory and in fieldwork. In this context, the non-random behavior of the correlation of these variables showed the establishment of several disease clusters in the studied area. This means that these neighborhoods present major risk factors for sporotrichosis transmission, due to its precarious infrastructure such as unpaved roads with exposed soil and the accumulation of solid waste that establishes a potential epidemiological scenario for sporotrichosis, as well as other infectious processes.^17^^,^^24^^,^^25^

The identification of the low supply of services and health centers and the low coverage of environmental surveillance in areas with quantitative significance of sporotrichosis cases points to the establishment of epidemiological silence, which limits the production of information used in the development process of public policies to mitigate the disease impacts. This situation can be also observed in several studies on the relationship between the availability of health services and the occurrence of infectious diseases, highlighting the weakness of the Brazilian Unified Health System, principally in the northern region of Brazil.^7^^,^^26^

The spatial dependence between neighborhoods that presented low and very low LCI and high and very high density of human sporotrichosis, as opposed to those with high LCI and absence of the disease constitutes a metaphor of the socioeconomic and epidemiological segregation existing in the city, due to these relationships be limited to specific areas. This fact also reflects the problems of the peripheral areas residents, who need the constitutional guarantee of citizenship concerning their right to access sanitary public services. That is because, according to the principles of equity, the distribution of health services and centers must obey a hierarchical logic for allocation of these resources, where demographic and socioeconomic indicators should define priorities.^27^ Therefore, social liabilities historically marked by non-sustainable development could be corrected.^7^

The fact of the spatial dependence between areas with a high density of human cases and the ecological niche of the great number of infected cats and precarious supply of health services and centers is related to the low socioeconomic conditions in which these populations are inserted, as observed with the use of the LCI. Thus, the geographic conjunction of these variables constitutes an active circuit for socioeconomic and environmental production of sporotrichosis, which points the health inequalities related to the concentration of primary health care for the disease in the central area of the city, where no cases were reported. This fact also indicates a socio-spatial segregation problem in Belém, based on the hierarchy of the population's access to public health services, because patients who live in peripheral neighborhoods far from the center must travel great distances to find health assistance.^7^^,^^18^

Thus, this situation puts a significant populational amount that lives in the city's peripheral AD in a lower social position regarding the difficulty in continuously maintaining the treatment prescribed for the disease, which means that many may abandon it, as observed in other infectious processes.^28^ It is important to consider the existence of high-income condominiums in these areas, whose inhabitants number is very low in comparison to the population of the peripheral neighborhoods that surround them. This means that the need for expanding public health policies to mitigate the disease occurrence both in humans and animals among the AD is imperative given the demographic context, as advocated by World Health Organization and the Brazilian Health Ministry in the global guidelines centered on the one-health dimension.^29^^,^^30^

The characteristics of the sporotrichosis risk factors in Belém point to the challenge and complexity of understanding its epidemiological scenario beyond the physical limits of the quantitative evidence of the land use and occupation associated with the significant spatial correlation between the environmental and socioeconomic problems, identified by the geoprocessing techniques used. This situation is also aggravated by the demography of the neighborhoods and AD with great numbers of cases, where the higher number of people residing bring pressure on the few health resources offered by the government, as may be observed in other regions of Amazon.^31^^,^^32^ These facts also suggest the need to develop qualitative analyses of the social inequalities observed in these territories, as they constitute conditionings for the disease establishment.

Given the above, it was observed a low sporotrichosis spatial density in Belém central neighborhoods and very high in peripheral ones related to a socioeconomic segregation existing in the city, with a significant gradient of its LCI associated to a spatial dependence between environmental and demographic risk factors. In addition, a low level of public health services supply in peripheral areas was also observed. All these situations reveal the production of a socio-spatial hierarchy of the population regarding their access to better living conditions, pointing to non-sustainable development of the city.

Although the city is not endemic for the disease, the fact that it occurs points to the need for discussing public policies related to one health, mainly regarding epidemiological and environmental surveillance focusing on controlling anthropozoonoses. The use of geoprocessing techniques was satisfactory for showing the spatial dependence between the variables analyzed in this work. Finally, we suggest the strengthening of surveillance actions for the disease to promote its mitigation. Given that the risk of territorial expansion constitutes an imminent danger for the health of Belém population and considering the possibility for an outbreak of the disease, we can view this situation as a metaphor for non-sustainable development of the city presented here that reflects the situation of social inequalities of the Brazilian Amazon.

## Conflicts of interest

The authors declare no conflicts of interest.
